# Geographical variation of cerebrovascular disease in New York State: the correlation with income

**DOI:** 10.1186/1476-072X-4-25

**Published:** 2005-10-21

**Authors:** Daikwon Han, Shannon S Carrow, Peter A Rogerson, Frederick E Munschauer

**Affiliations:** 1 Department of Social and Preventive Medicine, University at Buffalo, Buffalo, NY 14214 USA; 2 Department of Geography and National Center for Geographic Information and Analysis, University at Buffalo, Buffalo, NY 14261 USA; 3 Department of Neurology and Jacobs Neurological Institute, University at Buffalo, Buffalo, NY 14203 USA; 4 Department of Biostatistics, University at Buffalo, Buffalo, NY 14214 USA

## Abstract

**Background:**

Income is known to be associated with cerebrovascular disease; however, little is known about the more detailed relationship between cerebrovascular disease and income. We examined the hypothesis that the geographical distribution of cerebrovascular disease in New York State may be predicted by a nonlinear model using income as a surrogate socioeconomic risk factor.

**Results:**

We used spatial clustering methods to identify areas with high and low prevalence of cerebrovascular disease at the ZIP code level after smoothing rates and correcting for edge effects; geographic locations of high and low clusters of cerebrovascular disease in New York State were identified with and without income adjustment. To examine effects of income, we calculated the excess number of cases using a non-linear regression with cerebrovascular disease rates taken as the dependent variable and income and income squared taken as independent variables. The resulting regression equation was: *excess rate *= 32.075 - 1.22*10^-4^(*income*) + 8.068*10^-10^(*income*^2^), and both income and income squared variables were significant at the 0.01 level. When income was included as a covariate in the non-linear regression, the number and size of clusters of high cerebrovascular disease prevalence decreased. Some 87 ZIP codes exceeded the critical value of the local statistic yielding a relative risk of 1.2. The majority of low cerebrovascular disease prevalence geographic clusters disappeared when the non-linear income effect was included. For linear regression, the excess rate of cerebrovascular disease falls with income; each $10,000 increase in median income of each ZIP code resulted in an average reduction of 3.83 observed cases. The significant nonlinear effect indicates a lessening of this income effect with increasing income.

**Conclusion:**

Income is a non-linear predictor of excess cerebrovascular disease rates, with both low and high observed cerebrovascular disease rate areas associated with higher income. Income alone explains a significant amount of the geographical variance in cerebrovascular disease across New York State since both high and low clusters of cerebrovascular disease dissipate or disappear with income adjustment. Geographical modeling, including non-linear effects of income, may allow for better identification of other non-traditional risk factors.

## Background

Cerebrovascular disease disproportionately affects certain areas of the United States, including many areas within New York State [1]. Western New York, specifically, has excessive rates of cerebrovascular disease whereas other areas of New York State are seemingly under-affected. In recent years, researchers from the Department of Neurology at the State University of New York at Buffalo have analyzed age-, gender-, and race-adjusted cerebrovascular disease hospitalization data throughout New York State, focusing mostly at the regional and county levels. These analyses demonstrated that there are rates that are higher than those in other regions, such as Western New York State, and these data suggested that these geographic differences cannot be fully attributed to age, gender, and/or race.

Epidemiologic studies, including our own, that have explored causes of cerebrovascular disease among various populations, have historically focused on identifying associations between vascular disease and traditional risk factors. This research is well-founded since cerebrovascular disease, the third leading cause of death in the US, has long been associated with such traditional risk factors as hypertension, diabetes, elevated cholesterol, obesity, and tobacco use [2-4]. However, traditional risk factors and demographic characteristics such as age, gender, and race explain only some of the observed variance in vascular disease rates. In recent years non-traditional risk factors, such as income and education, have emerged as predictors of cerebrovascular disease. Unlike traditional risk factor prevalence, which is largely difficult to measure, socioeconomic risk factor prevalence is often known.

A number of studies have explored the relationships between vascular disease and socioeconomic risk factors and have identified associations [5-8]. Recent literature has shown that socioeconomic factors, and specifically income, are more determinative of vascular health than traditional risk factors and may, in fact, be the best predictors of vascular disease [6]. Socioeconomic factors have been shown to contribute directly to behavioral causes of vascular disease where studies have indicated a greater propensity for behavioral risk factors among persons who had not completed high school and among those who are unemployed or are employed in unskilled or low-paid positions [9]. A myriad of studies have focused on the many ways in which income can adversely affect health and have demonstrated evidence of an association between income and cerebrovascular disease [10-12]. Shi et al. maintain that income inequality and stroke mortality are related in that income inequality affects psychosocial factors that exacerbate stroke risk factors [10]. Some theories regarding this association contend that disparities in income and social status create appreciable strain that ultimately impairs one's health [13,14].

In addition, there is considerable geographic variations in cerebrovascular disease mortality across various geographical scales; in the US and world, and even within New York State [1,15,16]. Disease mapping and cluster analyses have been used in addressing public health concerns, especially when one is interested in identifying spatial patterns of disease [17,18] Furthermore, geographic clustering analyses can be used as exploratory tools to identify areas of elevated disease risk, and thus to provide hypotheses on causal relationships of disease mechanism and some clues for unknown etiologic studies. For example, recent studies of geographic clustering of breast cancer incidence and mortality rates in the Northeastern US found significant spatio-temporal clustering of breast cancer in the Northeast [19,20].

There is cause to believe that income, is contributing to the heretofore unexplained variance in disease rates carried by certain areas of New York State. We questioned if the rates generated during our previous work would change once income was accounted for. We utilized a new geographic analysis method to examine clustering of cerebrovascular disease and the non-linear effects of income, one that has not been previously applied in cerebrovascular disease and socioeconomic risk data analysis in New York State.

The purpose of this study was to 1) pursue a cross-disciplinary, innovative approach to identifying a significant nontraditional socioeconomic risk factor, 2) correlate this socioeconomic risk factor with the prevalence of cerebrovascular events at the ZIP code level in New York State in the year 2000, and 3) apply income-adjusted geographic clustering analyses to identify geographic patterns of cerebrovascular disease correlated with income within ZIP codes in New York State. We hypothesized that the novel approach of geographical cluster analysis, together with nonlinear regression that associated spatial distributions of income with cerebrovascular disease spatial distributions would enhance the power to predict event rates as compared to traditional risk factors. Such factors would have the power to facilitate the identification of high-risk ZIP codes or groups of ZIP codes for direct interventions, as well as low-risk ZIP codes or groups of ZIP codes for further exploration.

## Results

### Geographic clustering of cerebrovascular disease in New York State

**Figure 1:**
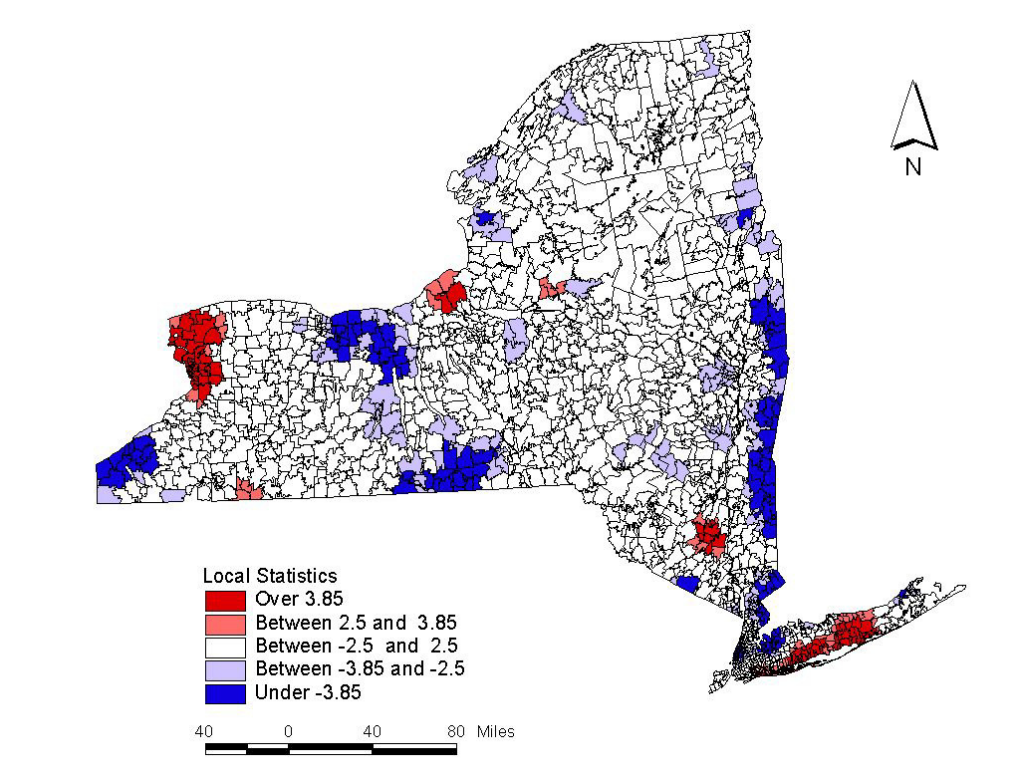
**Clustering of cerebrovascular disease in New York State**. Shown are map of New York State by zip code boundaries; areas of high prevalence of cerebrovascular disease are presented in red (local statistics over 3.85 and between 2.5 and 3.85), while areas of low prevalence are in blue (under -3.85 and between -2.5 and -3.85).

Figure 1, geographic clustering of cerebrovascular disease in New York State, shows the locations of clusters of significantly raised prevalence; (a) areas with local statistics greater than 3.85 are shown in dark red as statistically significant clusters that exceed the critical value, and (b) areas with local statistics greater than the commonly used threshold of 2.5 and less than 3.85 are highlighted in light red. The maximum local statistic was 9.39, and it was obtained in the center of the Buffalo-Niagara cluster (ZIP code 14224 in Erie County). One hundred ZIP code areas throughout New York State exceed the critical value; these areas contain 7,935 observed cases and 6,478.9 expected cases, yielding a relative risk of 1.23.

We also identified locations of significantly low prevalence; (a) areas with local statistics less than -3.85 are depicted in dark blue, and (b) areas with local statistics between -3.85 and -2.5 are shown in light blue. The minimum local statistic was -8.42 (corresponding to ZIP code 14892 in Chemung County). 153 ZIP code areas have local statistics below the critical value; these areas contain 5,412 observed cases and 7,161.3 expected cases, yielding a relative risk of 0.76. We also tested different kernel bandwidths, ranging from *σ *= 0.6 to *σ *= 2.5. These correspond to searching for clusters of different sizes; best results (in the form of highest local statistics) were obtained with *σ *= 1.0.

### Effects of income

To determine whether the differences between the observed and expected prevalence of cerebrovascular disease could be attributed to income, we performed a nonlinear regression; the age-adjusted cerebrovascular disease rate was taken as the dependent variable, and income and income squared were taken as independent variables. Although income has been previously noted as a risk factor, we also tested the hypothesis of a nonlinear effect of income. The resulting regression equation was:

*y *= 32.075 - 1.22*10^-4 ^(*income*) + 8.068*10^-10 ^(*income*^2^)

where *y *is the predicted age adjusted cerebrovascular disease rate, per 10,000 population. The value of *r*^2 ^is 0.045, but this is significantly different from zero, given the large number of ZIP codes examined. In addition, both the income and income squared variables were significant at the 0.01 level. As expected, the excess number of cases declines with increasing income. Each $10,000 increase in a ZIP code area's median income results in an average reduction in the age adjusted cerebrovascular disease rate of 1.22 per 10,000 individuals. In addition, the significant nonlinear effect indicates a lessening of the income effect with increasing income. For example, an increase in income from $20,000 to $30,000 results in a decrease in the cerebrovascular disease rate, on average, from 29.96 to 29.14 cases per 10,000 population while an increase from $60,000 to $70,000 results in a smaller decline – from 27.66 to 27.49 cases per 10,000 individuals. The age-adjusted rate is a minimum at an income level of $75,000; for ZIP codes with median income levels above this, the rate, on average, begins to increase.

### Clustering analysis with income adjustment

**Figure 2:**
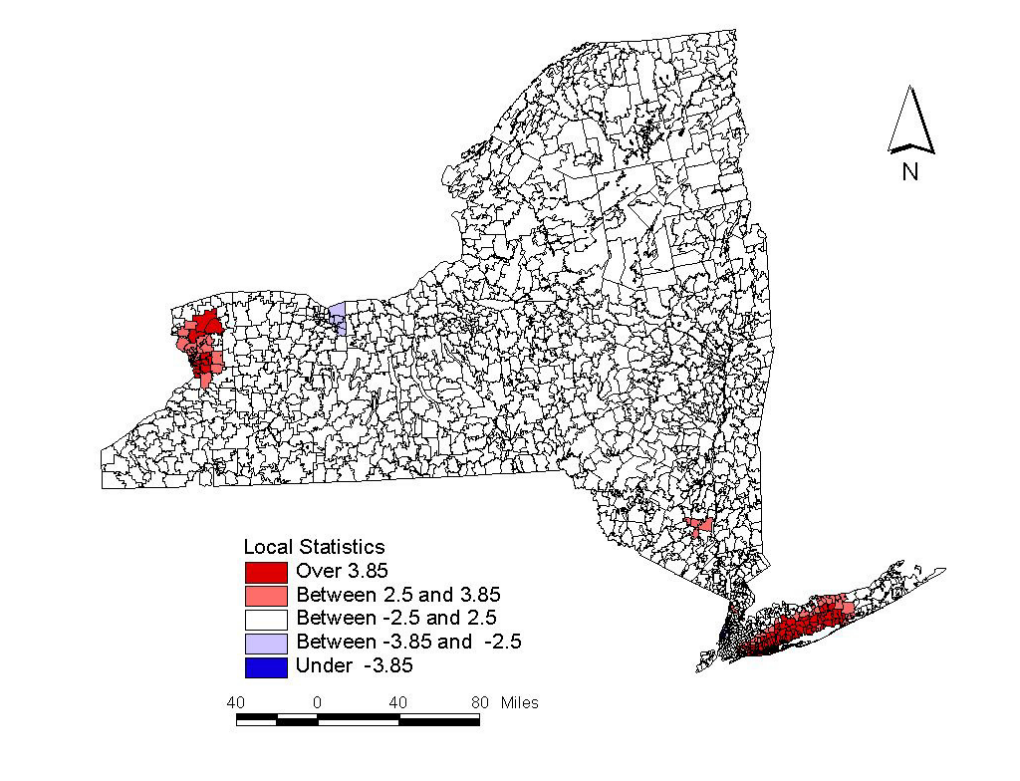
**Clustering of cerebrovascular disease with income adjustment**. Shown are map of New York State by zip code boundaries; areas of high prevalence of cerebrovascular disease with income adjustment are indicated in red (local statistics over 3.85 and between 2.5 and 3.85), while areas of low prevalence with income adjustment are in blue (under -3.85 and between -2.5 and -3.85).

Using the standardized residuals from the regression analysis, we identified geographic clusters of cerebrovascular disease in New York State with income adjustment. In this case the clusters in Figure 2 indicate areas of raised prevalence, once the effects of both age and income have been accounted for. The locations of high prevalence, with local statistics greater than the critical value of 3.85 are in dark red, and areas with local statistics greater than 2.5 and less than 3.85 are depicted in light red. There are two such clusters – one on Long Island and one in the Buffalo-Niagara area. The maximum local statistic was 7.57, and was obtained in the center of the Long Island cluster (ZIP code 11704). 87 ZIP code areas exceed the critical value; these contain 7,418 observed cases and 6,197.7 expected cases, yielding a relative risk of 1.2. Note that once income is included as a covariate, the number and size of clusters of high prevalence decreases; thirteen fewer ZIP code areas were significant after income adjustment.

**Figure 3:**
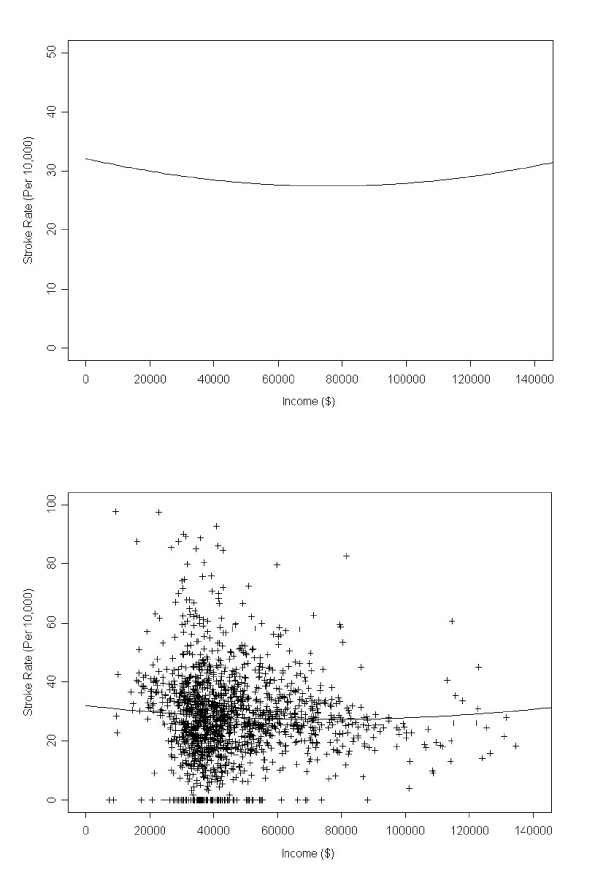
**Relationships between cerebrovascular disease rates and income**. Shown are resulting non-linear curves (top), and the curve with scatter plot of observed values (bottom).

We also identified the geographic locations of low prevalence after taking into account the non-linear effects of income. Again those areas with local statistics less than -3.85 in dark blue, and areas with local statistics between -3.85 and -2.5 in light blue are shown in the figure. The minimum local statistic was -4.42 (ZIP code 10069 in Westchester County). Thirteen ZIP code areas had local statistics below the critical value; these contained 917 observed cases and 1,262.3 expected cases, yielding a relative risk of 0.73. The majority of low prevalence areas in Figure 2 disappeared with income adjustment, indicating that many of the areas of low prevalence are explainable by income. Both the areas of high and low cerebrovascular disease prevalence identified are generally high income areas (the mean of the median incomes in the 87 high prevalence areas is $62,515, while the mean of the median incomes in the 13 low prevalence areas is $64,012), when compared to the New York State mean of the median income of $46,678. The resulting curves are presented in Figure 3. The scatterplot shows variation of cerebrovascular disease rates with income; shown are resulting non-linear curves (top), and the curve with scatter plot of observed values (bottom). There are some ZIP codes with zero observed cerebrovascular disease events.

## Discussion and conclusion

We found that income was statistically significantly associated with cerebrovascular disease prevalence after taking into account age. After adjusting for income, the relative risk of having cerebrovascular disease for residents of the Buffalo-Niagara and Long Island regions was 1.2 times greater than for residents in other areas. For residents of Westchester County, the relative risk for cerebrovascular disease was 0.73, inferring a protective effect of residence in that area after adjusting for age and income. The magnitude of the income effect in the nonlinear regression equation is small in part because the effects of income on prevalence are being averaged over the large number of individuals who live in each ZIP code area.

Initial analyses conducted with age but not income adjustment during this study corroborated findings from earlier works produced by our group. Of the one hundred ZIP code areas throughout New York that exceeded the critical value in the high clustering analyses without income, many of the ZIP codes are within western part, north-central part of New York State, and Long Island regions. The maximum local statistic of 9.39 was found in the center of the Buffalo-Niagara cluster. Also, in the analyses performed without income adjustment, highly concentrated areas of low clustering were found in the Finger Lakes area, namely Chemung County. The minimum local statistic was -8.42. This result is not unexpected since it substantiates previous findings as well.

Our analyses demonstrated that a number of areas of high and low disease prevalence of cerebrovascular disease are explainable by income when it is included as a covariate since the majority of clusters were absent when income adjustment was applied during nonlinear regression analysis. This supports our conviction that income, in fact, is a strong predictor of cerebrovascular disease. However, high clusters in the Buffalo-Niagara and Long Island regions remain above both the 3.85 and 2.5 critical values, as described earlier. As well, a significant low clustering area remains in the Hudson Valley region indicating that other factors are moderating low rates of cerebrovascular disease in this region.

Excess numbers of cerebrovascular disease cases decline with increasing income. Each $10,000 increase in a ZIP code's median income corresponds to a decrease in the rate of 1.22 per 10,000 population. This nonlinear effect weakens with increasing income and above about $75,600, the rate begins to increase slowly with income. The finding of high cerebrovascular disease prevalence in high income areas is unusual; one would expect this relationship to be inverse. Our study analyzed morbidity and not mortality data. Therefore, it is possible that residents in higher income areas survive longer with cerebrovascular disease than do those in lower income areas and deaths are not as prevalent.

There may be some bias related to spatial mismatch, since we have used zip-code level hospitalization data and ZCTA-level population and income data in our analysis. The US Census Bureau recently developed a new statistical entity, called ZCTA, to represent the United State postal service-defined zip code areas in a more cohesive way. Those ZCTA may be different from traditional zip codes, even though the ZCTA code equals the zip code in most cases [21]. We are well aware of the potential mismatch issue between the use of ZCTA and zip codes, especially in applying socio-economic data from the Census [22]. Unfortunately, we could not find any empirical study that validates this issue of spatial mismatch. As stated in the method section, our analyses were generally restricted to those areas that are found in both ZCTA and zip code tables. However, we were not able to assess the extent and scope of spatial mismatch, and the effects of such mismatch on the outcomes in this study.

Our study did not distinguish between types of cerebrovascular disease and therefore it is not known if income has a greater effect when correlated with one type of cerebrovascular disease than another. We do not know whether income is a causal factor or only a precipitating factor of cerebrovascular disease since we did not analyze individual-level data, nor did we adjust for other potential confounders. Based on our findings, it will be of great interest to further examine geographic distributions of traditional risk factors and non-traditional risk factors, such as education levels, occupation, measures of community deprivation, and environmental pollutants, to determine their contribution to geographic variations or clustering of cerebrovascular disease in New York State. In addition, it may be useful to examine other variables such as race and ethnicity to explore potential roles and relationships with those non-traditional risk factors.

In summary, income is a nonlinear predictor of cerebrovascular disease. Income alone explains a significant amount of the geographical variance in cerebrovascular disease across New York. These associations were observed after taking into account age. These findings support the contention that cerebrovascular disease cases are susceptible to the influence of socioeconomic factors, notably income. Where clusters failed to disappear, further analysis is indicated to determine what factors are implicated. Additional analysis may also be conducted to further explain the relationship with income. We suspect that a number of factors affect this relationship, including access to and utilization of care, and treatment patterns. These geographic analyses of multiple variables at the ZIP code level allow researchers to determine more precisely where disease events are occurring, along with the causative factors. Further analyses in other geographical scales, such as census tract level, other than at the ZIP code level may ensure these findings. This evidence-based information is necessary in order to affect public policy and isolate small areas such as ZIP codes or groups of ZIP codes for direct health interventions.

## Materials and methods

We obtained the Administratively Releasable (ADREL) inpatient hospitalization dataset for New York State from the Statewide Planning and Research Cooperative System (SPARCS) at the New York State Department of Health. Observed prevalence of cerebrovascular disease was extracted from the SPARCS inpatient dataset by ZIP code according to codes listed within "cerebrovascular disease" in the *International Classification of Diseases *(ICD). Income variables were extracted from US Bureau of the Census 2000 ZIP code level data files. For mapping purposes, ZIP code tabulation area (ZCTA) boundaries were also obtained from the US Bureau of the Census. Several ZCTAs were excluded for purposes of this study since they corresponded to hydrographic features such as lakes, parks, or forested lands. The final merged dataset that was prepared for geographic clustering analysis contained about 1600 ZCTAs, after restricting to those areas that are found in both ZCTA and zip code tables.

The cerebrovascular disease hospitalization rates were calculated using the principal diagnosis code issued at discharge that is included in each individual record within the SPARCS dataset. The following inpatient records from the SPARCS dataset were eliminated from the analyses: a) patients who lived out of state (N = 33,930), and b) patients who were discharged to another acute care hospital (N = 55,320). Note that the above numbers are not necessarily mutually exclusive. The ICD-9-CM codes used to determine cerebrovascular disease included ICD-9-CM code 430.00 through 438.99, cerebrovascular disease. The Census dataset provided population counts by gender and race in five-year age increments for each of the 1600 ZIP codes that had recorded populations. These five-year age groups were collapsed into the following 11 age groupings: 0–24, 25–34, 35–44, 45–54, 55–59, 60–64, 65–69, 70–74, 75–79, 80–84 and 85+. The 11 age groupings determined were appropriate for analysis of cerebrovascular disease; they were chosen to be wide enough to include a reasonably large population in each group, and they were narrow enough that the hospitalization rates would not vary too much within each age grouping.

Using age-specific population data from the Census, the age-adjusted expected number of cerebrovascular disease events was determined for each ZIP code using the indirect method of standardization. Age-adjustment allowed for a comparison without the influence of differences in how much older one population was than another. Several steps were required to obtain the age-adjusted hospitalization rates applying the indirect method of standardization [23]. The age-specific hospitalization rate by ZIP code for cerebrovascular disease was obtained first, where the numerator (age-specific counts of hospitalizations) was obtained from the SPARCS dataset, and the denominator (the age-specific population) was obtained from the US Census dataset. The SPARCS age-specific rate was multiplied by the age-specific Census counts of the population in each ZIP code to calculate the age-specific expected number of hospitalizations in the ZIP code. The total number of hospitalizations expected within each ZIP code was calculated by adding the expected number of hospitalizations across all age groups in the ZIP code.

The standardized rate (SR) for each ZIP code was then calculated as the ratio of the total number of hospitalizations observed in the ZIP code (O) divided by the total number of hospitalizations expected in the ZIP code (E), and the standard error of SR was calculated by applying the formula SE^2^(SR) = O/(E^2^). The percentage of excess risk (R) was calculated as SR-1.0, with R having the same standard error as SR. The 95% confidence interval for R was calculated as the interval from R-1.96SE to R+1.96SE.

### Spatial clustering methods

To test for the existence of geographic clusters of cerebrovascular disease exhibiting significantly higher or lower observations than could be expected upon the basis of age-structure, we used the statistical test suggested by Rogerson [24]. This approach has an attractive feature where the multiple testing associated with examining many ZIP codes is accounted for (otherwise, one might find "significant" areas of raised prevalence, but only because so many different geographic areas were being examined). In this sense it is similar to the spatial scan statistic popularized by Kulldorff [25]. We first transformed the observed and expected number of cases into a standardized, normal score for each ZIP code. Specifically, the quantity  will have an approximate normal distribution, with mean 0 and variance 1, where *O *is the observed number of cases, and *E *is the expected number of cases in ZIP code *i*. To optimize the detection of geographic clusters of a given size, these standardized scores need to be smoothed, by calculating for each ZIP code a *z*-score (*z*_*i*_) that is a weighted sum of the scores in the geographic neighborhood of the ZIP code:



where the weights are large near the ZIP code, and get smaller with distance:



and where *d*_*ij *_is the distance between the centroids of ZIP code areas *i *and *j*, and *σ *is a parameter indicating how quickly the weights change with distance (this can be thought of, approximately, as the radius of the neighborhood around each ZIP code).

The *z*_*i *_scores are known as local statistics, and they also each have a normal distribution with mean 0 and variance 1. If the null hypothesis of no geographic clustering is true, 95% of the time a map of the *z *scores will have a maximum that is no larger than:



where *A *is the number of subareas (1600). In our case, we used *σ *= 1, corresponding to defining, for each ZIP code, a neighborhood of approximately one ZIP code area in each direction. This yields a critical value of z* = 3.85.

An additional step is taken to correct for edge effects before carrying out the weighting described above (areas near the edge of the map must be treated differently, since they do not have as many neighboring ZIP code areas to carry out the smoothing). We began by overlaying a square grid containing lattice points at intervals equal to 5.5 miles (which is the median distance between ZIP code centroids) onto the study area. We then created additional, hypothetical ZIP code centroids around the border of New York State, and assigned them hypothetical, standardized scores, in keeping with the null hypothesis that there was no raised prevalence in these hypothetical locations.

We conducted a similar analysis of geographic clustering after adjusting for income in each ZIP code area. In this case, a regression analysis was carried out by first assuming a quadratic relationship between the excess number of cerebrovascular cases in a ZIP code area and income. We then used the standardized regression residuals (which has a normal distribution with mean 0 and variance 1) as input into the geographic clustering analysis. These geographic clustering analyses were carried out using S-Plus and exported into ArcView GIS for visualization.

## Authors' contributions

DH and SSC performed statistical and spatial clustering analysis, and wrote the manuscript. PAR and FEM provided critical review and input. DH, SSC, PAR and FEM participated in the design of the study, participated in interpretation, as well as in data acquisition efforts. All authors read and approved this manuscript.
